# Myopia Control with Low-Dose Atropine in European Children: Six-Month Results from a Randomized, Double-Masked, Placebo-Controlled, Multicenter Study

**DOI:** 10.3390/jpm13020325

**Published:** 2023-02-14

**Authors:** Anders Hvid-Hansen, Nina Jacobsen, Flemming Møller, Toke Bek, Brice Ozenne, Line Kessel

**Affiliations:** 1 Department of Ophthalmology, Copenhagen University Hospital—Rigshospitalet-Glostrup, DK-2600 Glostrup, Denmark; 2 Department of Clinical Medicine, University of Copenhagen, DK-2200 København N, Denmark; 3 Department of Ophthalmology, University Hospital of Southern Denmark—Vejle Hospital, DK-7100 Vejle, Denmark; 4 Department of Ophthalmology, Aarhus University Hospital, DK-8200 Aarhus N, Denmark; 5 Department of Public Health, Section of Biostatistics, University of Copenhagen, DK-1014 København K, Denmark; 6 Neurobiology Research Unit, Copenhagen University Hospital—Rigshospitalet, DK-2200 København N, Denmark

**Keywords:** myopia, myopia control, low-dose atropine, axial length, spherical equivalent

## Abstract

The effect and safety of low-dose atropine in myopia control have not been studied in randomized, placebo-controlled trials outside Asia. We investigated the efficacy and safety of 0.1% atropine loading dose and 0.01% atropine compared with a placebo in a European population. Investigator-initiated, randomized, double-masked, placebo-controlled, equal-allocation, multicenter study comparing 0.1% atropine loading dose (six months) followed by 0.01% atropine (18 months), 0.01% atropine (24 months), and placebo (24 months). Participants were monitored for a 12-months washout period. Outcome measures were axial length (AL), cycloplegic spherical equivalent (SE), photopic and mesopic pupil size, accommodation amplitude, visual acuity, intraocular pressure (IOP), and adverse reactions and events. We randomized 97 participants (mean [standard deviation] age, 9.4 [1.7] years; 55 girls (57%) and 42 boys (43%)). After six months, AL was 0.13 mm shorter (95% confidence interval [CI], −0.18 to −0.07 [adjusted *p* < 0.001]) with 0.1% atropine loading dose and 0.06 mm shorter (95% CI, −0.11 to −0.01 [adjusted *p* = 0.06]) with 0.01% atropine than in the placebo group. We observed similar dose-dependent changes in SE, pupil size, accommodation amplitude, and adverse reactions. No significant differences in visual acuity or IOP were found between groups, and no serious adverse reactions were reported. We found a dose-dependent effect of low-dose atropine in European children without adverse reactions requiring photochromatic or progressive spectacles. Our results are comparable to those observed in East Asia, indicating that results on myopia control with low-dose atropine are generalizable across populations with different racial backgrounds.

## 1. Introduction

Myopia is one of the most common eye disorders worldwide, and uncorrected myopia is the leading cause of distance visual impairment globally [1]. The prevalence of myopia is increasing, especially in urban areas of East Asia, where 80–90% of those finishing secondary school are myopic [2]. Western prevalences are lower, with approximately one-third of the adult population being myopic in Europe and the United States [3,4,5,6]. 

Myopia, particularly high myopia (−6 diopters [D] or more), increases the risk of sight-threatening eye diseases, including glaucoma, retinal detachment, myopic maculopathy, and myopic choroidal neovascularization [7,8,9]. These severe complications highlight why myopia should not be dismissed as a benign eye condition correctable with glasses, contact lenses, or refractive surgery but rather considered an ocular health issue that eye care professionals need to diagnose, treat, and prevent. 

The risk of developing ocular complications correlates to myopic elongation of the eye, which has sparked an interest in developing interventions to reduce the pathological growth of the eyeball [10]. The field is rapidly evolving and includes behavioral, optical, and pharmacological approaches [11]. 

The Atropine for the Treatment of Myopia 1 and 2 (ATOM 1 and ATOM 2) and the Low-Concentration Atropine for Myopia Progression (LAMP) studies demonstrate a dose-dependent response of topical atropine eye drops in reducing myopia progression in Asian children [12,13,14]. Additionally, the number of side effects (e.g., photophobia and blurred near vision) and rebound (i.e., faster myopia progression upon treatment cessation) also depict a dose dependency [12,13,14,15]. Overall, the beneficial balance between efficacy and safety has led to the widespread use of low-dose atropine as a promising treatment option for myopia control, especially in East Asia [16]. 

To date, the effect of low-dose atropine on myopia progression has been extensively studied in East Asia, whereas the effect and safety outside Asia are largely uninvestigated in randomized trials [17]. Racial differences in atropine effectiveness and sensitivity may exist due to different levels of pigmentation in the iris, and adverse reactions may be more prevalent in White populations with lighter irides [14,18].

As a result, we conducted the Low-dose Atropine for the Prevention of Myopia Progression in Danish Children (APP) study to assess the efficacy and safety of 0.1% atropine loading dose and 0.01% atropine alone compared to placebo. The primary hypotheses were (1) 0.01% atropine is safe and effective in reducing myopia progression, (2) 0.1% atropine loading dose is safe and superior to 0.01% atropine alone, and (3) the rebound effect is clinically insignificant at dose change and treatment cessation. To our knowledge, no other studies have compared the 0.01% atropine and 0.1% atropine loading dose to a placebo in a European population. This article provides the baseline characteristics of the enrolled participants, describes the study’s methods, and presents the results from six months follow-up.

## 2. Materials and Methods

### 2.1. Design and Intervention

The present study is an ongoing investigator-initiated, 36-month, double-masked, placebo-controlled, multicenter, equal-allocation randomized study designed to evaluate the efficacy and safety of topical 0.1% atropine loading dose and 0.01% atropine alone eye drops in reducing myopic progression in Danish children. The study was conducted at Copenhagen University Hospital—Rigshospitalet, Aarhus University Hospital, and University Hospital of Southern Denmark—Vejle Hospital. In phase 1 (treatment phase), the participants were randomized to receive either a 0.1% atropine loading dose for six months followed by 0.01% atropine for 18 months, 0.01% atropine for 24 months or placebo for 24 months. The trial medication was administered as one eye drop daily in each eye at bedtime. In phase 2 (washout phase), treatment was stopped, and participants were monitored for 12 months. 

Trial medication was prepared by Skanderborg Apotek, Denmark, following good manufacturing practices. Benzalkonium chloride was used as a preservative. At each delivery, the compounding pharmacy provided batch certificates with a declaration of sterility, concentration, and stability.

### 2.2. Participants and Sample Size

We recruited participants among subjects referred by ophthalmologists and optometrists. Self-referral by parents was also accepted. 

Inclusion criteria were age 6 to 12 years; myopia (spherical component by cycloplegic autorefraction in at least one eye) of ≤−1 D if age was ≥6 to <9 years, or ≤−2 D if age was ≥9 to ≤12 years; and astigmatism of less than −1.5 D. 

Exclusion criteria were ocular pathology (e.g., amblyopia, strabismus, and keratoconus), myopia related to retinal dystrophies, connective tissue disorders (e.g., Ehlers Danlos syndrome, Marfan syndrome, and Sticklers syndrome), previous eye surgery, previous use of agents thought to affect myopia progression (e.g., atropine, pirenzepine or 7-methylxanthine), previous use of soft bifocal/multifocal or orthokeratology contact lenses, known allergy to atropine or any of the contents of the trial medication, non-compliance to eye examinations, serious systemic health troubles (e.g., cardiac or respiratory illness), and developmental disorders and delays. 

The primary endpoint was the mean change in axial length (AL) 36 months after baseline. Because no valid data for AL elongation in Danish myopic children were available at the time of the planning and AL is strongly related to the refraction of the eye, the power calculation was performed using the refraction (in spherical equivalent [SE]) as a surrogate measure. A −1.2 ± 0.69 D progression during two years in untreated childhood myopia has been shown in Asian children [12]. A comparable myopia progression rate of −1.14 ± 0.69 D has been documented in Danish school children wearing single-vision spectacles [19]. Given a significance level of 0.05 and a statistical power of 80%, we estimated that 21 subjects per group were needed to detect a difference in myopia progression of 50% after 24 months. To compensate for the study length, dropout rates, and unknown effect of low-dose atropine in non-Asian children, 50 subjects per group were initially planned to be enrolled. However, because of a more extended recruitment period than expected due to the COVID-19 pandemic, fewer dropouts than anticipated, and a fair margin to group sizes of 21 subjects, the recruitment was terminated before the total number of 150 subjects was enrolled.

### 2.3. Randomization and Masking

At baseline, eligible participants were randomized to one of the three interventional groups. We used the randomization instrument in Research Electronic Data Capture (REDCap) hosted at Capital Region, Denmark [20,21]. Before study initiation, an independent researcher created and uploaded an allocation list with unique randomization codes to REDCap. The list was created using https://www.sealedenvelope.com/simple-randomiser/v1/lists (accessed on 2 January 2023). Block sizes were 3 and 6, the list length was 450, and the stratification factor mirrored study sites. The independent researcher stored the allocation list and distributed it to the compounding pharmacy. Moreover, the sponsor-investigator (L.K.) received and stored a sealed envelope for each randomization code containing information on the specific treatment. All envelopes were kept under lock and were inaccessible to other study personnel. The randomization code was the only identifying feature on the packages and bottles with trial medication. 

Allocation concealment was maintained by masking investigators, key personnel performing the ocular measurements, parents, and participants to randomization status throughout the entire study period.

### 2.4. Examinations and Outcomes

A screening visit was performed to assess the eligibility of potential participants. Participants who fulfilled the inclusion criteria and none of the exclusion criteria were invited to participate in the study. To maximize treatment adherence to daily eye drop administration, consenting participants and parents were given the choice of a trial period of at-home administration of lubricant eye drops (Viskøse Øjendråber “Ophtha”, Hypromellose 3.5 mg/mL, Actavis Group PTC ehf., Hafnarfjordur, Iceland). Participants were reassessed at the baseline visit (randomization and treatment start) and again at 3, 6, 9, 12, 18, 24 (treatment stop and washout start), 30, and 36 months. If the period between screening and baseline was less than four weeks, some examinations were optional at the baseline visit (Table S1). 

At each visit, AL was measured using a swept-source optical coherence tomography (SS-OCT)-based biometer IOLMaster 700 (Carl Zeiss Meditec AG, Jena, Germany). Central corneal thickness (CCT), anterior chamber depth (ACD), and lens thickness (LT) were also obtained from the IOLMaster 700. Corneal tomographic data was measured by Scheimpflug imaging (Pentacam HR, Oculus Optikgeräte GmbH, Wetzlar, Germany). Mesopic and photopic pupil sizes were measured using the DP-2000 Human Laboratory Pupillometer (NeurOptics, Irvine, CA, USA) with stimulus intensities of 4 lux and 300 lux, respectively. At least five pupil size measurements (with a maximum tolerability range of 1.0 mm) were performed for each setting. Choroidal thickness, optic nerve head characteristics, and macular angiography were assessed by SS-OCT using the DRI OCT Triton (Topcon Europe Medical BV, Capelle aan den IJssel The Netherlands). Thickness and volume measurements were obtained from the built-in software (IMAGEnet 6, Topcon Europe Medical BV, The Netherlands). Intraocular pressure (IOP) was assessed using a rebound tonometer (iCare TA01i, Icare Finland Oy, Vantaa, Finland) with an average of a minimum of 5 readings. The near point of accommodation was measured using a Royal Air Force (RAF) near point rule (Good-lite, Elgin, IL, USA) with the best-corrected distance spectacle correction. The near point of accommodation was calculated as the average of push-up and push-down values, and the accommodation amplitude was calculated as the inverse of the average near point. Distance best-corrected visual acuity (BCVA) logarithm of the minimum angle of resolution (logMAR) was examined by using HOTV charts (HOTV series ETDRS (4 m), Precision Vision Inc., Woodstock, IL, USA). Near visual acuity was assessed using best-corrected distance spectacle correction with a logMAR near vision HOTV chart (HOTV (40 cm), Precision Vision Inc., USA). Non-cycloplegic and cycloplegic autorefraction were assessed with the Retinomax K-plus 3 (Right Mfg. Co. Ltd., Tokyo, Japan) handheld autorefractor. In both cases, the average of five readings was calculated with a predefined quality cut-off score of ≥7. Cycloplegic autorefraction was assessed 30 min after the last of 2 drops of cyclopentolate 1% (Minims Cyclopentolate Hydrochloride 1%, Bausch & Lomb Nordic AB, Stockholm, Sweden) was administered to both eyes at 5 min apart. If pupillary light reflex was still present or the pupil size was less than 6 mm after 30 min, additional eye drops were administered, followed by a 30 min wait before cycloplegic measurement. SE was calculated as spherical power plus half-cylinder power. Cycloplegic autorefraction was the only measurement performed under cycloplegia. An examiner graded the iris color, and the parents reported the participant’s ethnicity. 

Participants were prescribed best-corrected spectacles at all visits unless the difference between their spectacles and the newly recorded correction was <0.5 D and/or BCVA was ≥0.80 with their spectacles [19]. Participants were offered photochromatic glasses (darken on exposure to ultraviolet or sunlight) or progressive glasses (reading add) if they experienced problems with glare or near vision, respectively [16,22]. The study covered expenses up to an annual maximum of 1000 DKK (approximately 150 USD) for refractive correction. Monofocal spectacles, contact lenses, or both were acceptable.

Compliance was evaluated using at-home administered charts with boxes for ticking daily trial medication use. Using this method, a compliance rate of 75% (i.e., a mean of 5.25 days/week) was considered compliant [22,23].

During each visit, participants and parents were given an opportunity to report any medical illness or side effects. They were also explicitly asked about symptoms related to visual disturbance, glare, blurred near vision, allergy, and if participants had been ill or hospitalized since the last visit. Investigators assessed the distinction between adverse reactions and events.

### 2.5. Approvals

The study was registered in the European Union Drug Regulating Authorities Clinical Trials Database (EudraCT: 2018-001286-16) and at www.clinicaltrials.gov (accessed on 2 January 2023) (NTC no.: NCT03911271) before initiation. The study was approved by the Committee on Health Research Ethics for the Capital Region of Denmark (reference no.: H-18043987), the Danish Medicines Agency (reference no.: 2018-040088), and The Danish Data Protection Agency (reference no.: P-2022-85). 

The study sites were monitored according to the GCP quality standards by the GCP units at Copenhagen University Hospital, Aalborg and Aarhus University Hospitals, and Odense University Hospital. The study was conducted following the tenets of the Declaration of Helsinki. Written informed consent was obtained from parents, and verbal assent was obtained from children before the examination at the screening visit.

### 2.6. Statistical Analysis

All statistical analyses were conducted according to a prespecified analytical plan using the R statistical software version 4.1.0 (R Program for Statistical Computing, Vienna, Austria) [24] and the LMM-star package (R package version 0.8.10, Copenhagen, Denmark) [25]. Measurements obtained from both eyes were averaged for all analyses. Pairwise comparisons of continuous data between the placebo and interventional groups were performed using a linear mixed model with an effect of treatment and study site in the mean structure and an unstructured covariance pattern for modeling the variability and dependency between measurements from the same patient. Since all patients were treatment-free at baseline, the mean of the linear mixed model was constrained to be the same in all treatment groups [25]. Dichotomous outcomes were analyzed with Fisher’s exact test. All hypothesis tests were two-sided. Primary analyses (i.e., change in AL and SE at 36 months compared to placebo) were planned to be adjusted for multiple testing using Bonferroni correction. All secondary analyses were adjusted for multiple testing using a false discovery rate (FDR) correction [26]. An adjusted *P* (adj-*P*) value less than 0.05 was considered statistically significant. Missing data were implicitly handled by maximum likelihood estimation in the constrained linear mixed model. The independent researcher masked statistical analyses by renaming the study ID and the interventional groups.

## 3. Results

**Figure 1:**
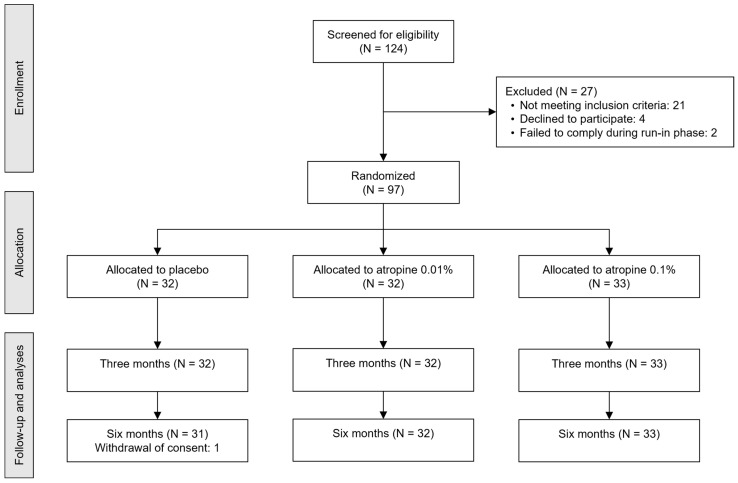
CONSORT diagram for the flow of participants. **Notes:** The run-in phase consisted of at-home administration of lubricant eye drops to access and maximize future treatment adherence. **Abbreviations:** CONSORT = consolidated standards of reporting trials; N = number of participants.

We screened 124 subjects for eligibility between May 2019 and April 2021. Ninety-seven participants (mean [standard deviation (SD)] age, 9.4 [1.7] years; 55 girls (57%) and 42 boys (43%)) were randomized, with 33, 32, and 32 participants in the 0.1% atropine loading dose, 0.01% atropine alone, and placebo groups, respectively (Table 1). During the six-month follow-up, 1 participant withdrew from the placebo group due to parental concerns related to potential side effects (Figure 1). At both 3 and 6 months follow-up, all participants reported compliance rates above 75% (Table S2).

### 3.1. Changes in AL and SE

**Figure 2:**
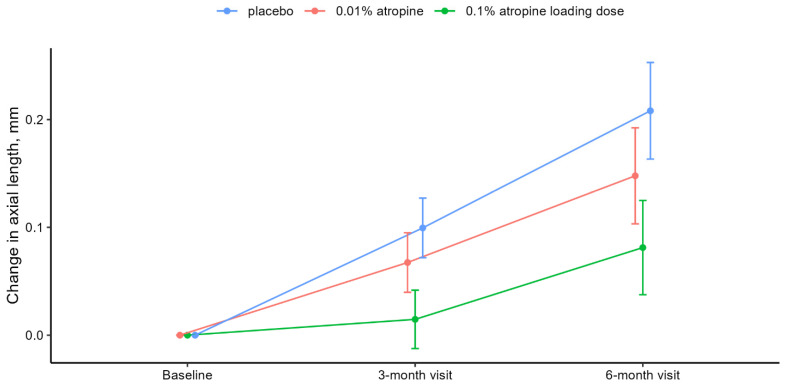
Change in AL from baseline. **Notes:** Mean change in axial length from baseline to six-month visit with placebo (blue), 0.01% atropine (red), and 0.1% atropine loading dose (green). Error bars denote the 95% CIs of the mean changes. Means and CIs are derived from the constrained linear mixed model with inherent baseline adjustment. *P* and adj-*P* values are presented in Table 2. **Abbreviations:** adj-*P* = adjusted *P*; CI = confidence interval.

AL and SE showed a concentration-dependent development with the largest change in the placebo group. At six months, the mean elongation of AL was 0.08 mm (95% confidence interval [CI], 0.04–0.12), 0.15 mm (95% CI, 0.10–0.19), and 0.21 mm (95% CI, 0.16–0.25) in the 0.1% atropine loading dose, 0.01% atropine, and placebo groups, respectively. Differences between the placebo group and interventional groups were −0.06 mm (95% CI, −0.11 to −0.01 [adj-*P* = 0.06]) with 0.01% atropine and −0.13 mm (95% CI, −0.18 to −0.07 [adj-*P* < 0.001]) with 0.1% atropine loading dose. Table 2 and Figure 2 present the constrained linear mixed model results.

A hyperopic shift of +0.15 D was observed in the 0.1% atropine loading dose group at three months but not in the 0.01% atropine group. At six months, the mean change in SE was +0.03 D (95% CI, −0.11 to 0.18) D, −0.21 D (95% CI, −0.35 to −0.06), and −0.37 D (95% CI, −0.52 to −0.21) in the 0.1% atropine loading dose, 0.01% atropine, and placebo groups, respectively. Pairwise comparison showed a statistically significant difference between the 0.1% atropine loading dose group and placebo of 0.40 D (95% CI, 0.22–0.57 [adj-*P* < 0.001]), while the difference between the 0.01% atropine group and placebo of 0.16 D (95% CI, −0.02 to 0.34 [adj-*P* = 0.16]) was not statistically significant.

### 3.2. Changes in Pupil Diameter, Accommodation, Visual Acuity, and IOP

Changes in photopic and mesopic pupil size also followed a concentration-dependent response, and differences between placebo and the treatment groups were stable from three to six months. Under photopic conditions, the pupil size increased significantly by 1.83 mm (95% CI, 1.58–2.09 [adj-*P* < 0.001]) in the 0.1% atropine loading dose group compared to the placebo. In comparison, the difference between the 0.01% atropine group and placebo (0.20 mm [95% CI, −0.06 to 0.45]) was not statistically significant (adj-*P* = 0.23). 

Mesopic pupil size increased significantly in both interventional groups compared to placebo by 2.18 mm (95% CI, 1.84–2.52 [adj-*P* < 0.001]) in the 0.1% atropine loading dose group and 0.42 mm (95% CI, 0.08–0.76 [adj-*P* = 0.04]) in 0.01% atropine group. 

We found a significant difference in accommodation amplitude between 0.1% atropine loading dose and placebo (−4.88 D [95% CI, −6.37 to −3.38; adj-*P* < 0.001]), but no significant difference between 0.01% atropine and placebo (adj-*P* = 0.75). 

Changes in distance BCVA, near BCVA, and IOP were small, and pairwise comparisons between groups did not show clinically significant differences.

### 3.3. Photophobia, Blurred near Vision, and Other Adverse Events

There were no significant differences in the proportions of adverse reactions and events between the 0.01% atropine and placebo groups (Table 3). In the 0.1% atropine loading dose group, the proportions of participants and parents that reported adverse reactions of mydriasis, photophobia, and blurred near vision were significantly higher than in the placebo group. Although the numbers reduced over time, the differences remained statistically significant. All adverse reactions were graded mild, and no participant requested a prescription for photochromic or progressive spectacles. A further detailed reporting of adverse reactions and events was not possible due to the intention to maintain masking. Adverse events included three participants reporting localized skin changes, two participants having intermittent abdominal pain for a few days, and one case each of otitis media, sleep disturbance, and a fractured toe. One participant was hospitalized for meningitis observation but discharged the same day without further follow-up. There were no serious adverse reactions reported.

## 4. Discussion

This randomized placebo-controlled trial demonstrates the efficacy and safety of 0.1% atropine loading dose, and 0.01% atropine alone eye drops to reduce myopic progression in a European population. After six months, AL had increased 0.13 mm (61%) less in the 0.1% atropine loading dose and 0.06 mm (29%) less in the 0.01% atropine groups compared to the placebo group. To our knowledge, this is the first randomized placebo-controlled trial to show a concentration-dependent effect of atropine in slowing myopic progression in a European population. 

The dose dependency was also evident in the changes in photopic and mesopic pupil size, accommodation loss, and the frequency of adverse reactions, with significantly higher rates in the 0.1% atropine loading dose group than in the placebo group. The LAMP group recently suggested that less-pigmented irides in White populations might be more prone to atropine-induced side effects [14]. However, photochromatic spectacles or near-vision add were not requested by any participants, and there were no differences between groups in distance or near visual acuity.

The effect and safety of low-dose atropine on reducing myopia progression are well-documented in multiple randomized clinical trials in Asian children [13,14,27,28,29,30,31,32], whereas the use of low-dose atropine in myopic populations in Europe and the United States has not been investigated in randomized, placebo-controlled trials until date [33,34,35,36,37]. Our study indicates that 0.1% and 0.01% atropine have similar effects and dose-dependency in slowing AL elongation and SE progression in European and Asian children. We observed comparable progression in AL and SE in the 0.01% atropine and placebo groups at six months, as the LAMP study did at 4 and 8 months [23]. We found no statistically significant treatment effect on AL or SE progression in the 0.01% atropine group compared to the placebo, similar to the LAMP study [23]. Additionally, our 0.1% atropine loading dose demonstrated less myopic progression than the 0.05% atropine group from the LAMP study, supporting the concentration-dependent effect [23].

Direct comparison of changes in pupil size is difficult because studies have used different methods. We used a dedicated dynamic pupillometer, reporting six-month changes of 1.83 mm and 0.20 mm under photopic conditions in the 0.1% atropine loading dose and 0.01% atropine, respectively, with similar changes over time and under mesopic conditions. In ATOM 2, the distinction between the baseline and second baseline showed that most of the 12-month changes in pupil size and accommodation amplitude occurred in the first 14 days after treatment started [22]. With this in mind, the 12-month results showed that pupil size changes were more pronounced in the ATOM 2 and LAMP studies, and the latter also reported bigger photopic changes than mesopic changes [16,22]. 

We found an accommodation loss of 7.0 D, 2.5 D, and 2.1 D in the 0.1% atropine loading dose, 0.01% atropine, and placebo groups, respectively. ATOM 2 reported higher reductions of 10.9 D and 4.4 D in the 0.1% atropine and 0.01% atropine, respectively, while LAMP presented a smaller reduction of 0.3 D in both the 0.01% atropine and placebo [22,23]. These results were all obtained using the RAF rule, and some variability may reflect the semi-objective nature of the procedure [14]. However, our findings do not suggest that low-dose atropine causes more pronounced mydriasis or accommodation loss in European children when compared to previously reported changes in Asian children [22,23]. Distance and near visual acuity were not affected by changes in pupil size and accommodation in our study. This is consistent with previous suggestions that patients will not experience symptoms during daily visual activities if they have less than 3 mm of pupil dilation under photopic conditions and accommodation of 5 D, which should be compared to 9.4 D, 13.9 D, and 14.3 D in the 0.1% atropine loading dose, 0.01% atropine, and placebo groups, respectively, in our study [38]. 

There are some limitations to our study. First, the COVID-19 pandemic made the inclusion period longer than expected, leading the recruitment to stop before the anticipated number of participants was reached. However, only one participant withdrew from the study, and we kept an acceptable margin to the calculated group size of 21 participants. Second, our inclusion criteria did not include a documented myopic progression. Instead, we used two age-dependent myopia cut-offs. Third, our comprehensive investigations, including ocular biometry, cycloplegic refraction, pupil size, accommodation amplitude, visual acuity, and retinal imaging, were very time-consuming and did not allow for resting periods between measurements, e.g., pupil size and choroidal thickness, considering the age and cooperation of the participants. Fourth, we did not assess the reproducibility of the devices used and the possible impact of ocular diurnal rhythms [39]. Future research on these aspects in myopic children is warranted. 

In conclusion, this study demonstrates that 0.1% and 0.01% atropine show similar effects and dose-dependency in reducing myopic progression in European children after six months of treatment, compared to previous reports from East Asia. In addition, there appear to be no racial differences in atropine-induced mydriasis and loss of accommodation. Our findings indicate that results on myopia control with low-dose atropine are generalizable across populations with different racial backgrounds.

## Figures and Tables

**Table 1:** Baseline characteristics of randomized participants.

		Treatment Groups		
Characteristic	All	Placebo	Low-Dose (0.01% Atropine)	Loading Dose (0.1% to 0.01% Atropine)
N (%)	97	32 (33.0)	32 (33.0)	33 (34.0)
Site, N (%)				
Copenhagen	43 (44.3)	14 (32.6)	14 (32.6)	15 (34.9)
Vejle	41 (42.3)	13 (31.7)	14 (34.1)	14 (34.1)
Aarhus	13 (13.4)	5 (38.5)	4 (30.8)	4 (30.8)
Female/Male (%)	55/42 (57/43)	18/14 (56/44)	18/14 (56/44)	19/14 (58/42)
Age, mean (SD), years	9.4 (1.7)	9.2 (1.6)	9.4 (1.9)	9.5 (1.5)
Race, N (%)				
White	82 (84.5)	29 (90.6)	25 (78.1)	28 (84.8)
Mixed race	9 (9.3)	3 (9.4)	3 (9.4)	3 (9.1)
Middle East	3 (3.1)	―	―	―
Asian	2 (2.1)	―	―	―
Black or African American	1 (1.0)	―	―	―
AL, mean (SD), mm	24.48 (0.84)	24.41 (0.90)	24.56 (0.78)	24.48 (0.86)
SE, mean (SD), D	−3.02 (1.27)	−3.07 (1.04)	−2.97 (1.13)	−3.0 (1.59)
CCT, mean (SD), µm	546.8 (30.3)	546.0 (35.1)	546.4 (25.7)	547.8 (30.3)
ACD, mean (SD), mm	3.31 (0.24)	3.32 (0.27)	3.33 (0.26)	3.28 (0.19)
Photopic pupil size, mean (SD), mm	2.68 (0.33)	2.64 (0.31)	2.60 (0.30)	2.80 (0.36)
Mesopic pupil size, mean (SD), mm	4.24 (0.74)	4.11 (0.74)	4.17 (0.58)	4.42 (0.84)
Distance BCVA, mean (SD), logMAR	−0.09 (0.06)	−0.09 (0.06)	−0.09 (0.05)	−0.08 (0.07)
Near BCVA, mean (SD), logMAR	−0.04 (0.09)	−0.03 (0.09)	−0.05 (0.09)	−0.04 (0.09)
Accom. amplitude, mean (SD), D	14.5 (4.0)	14.1 (4.4)	14.3 (3.9)	15.1 (3.6)
ChT, mean (SD), µm	247.9 (66.2)	244.0 (65.1)	259.8 (66.7)	240.2 (67.2)
IOP, mean (SD), mmHg	16.1 (2.7)	16.1 (2.5)	15.8 (3.1)	16.5 (2.7)

**Abbreviations**: Accom. = accommodation; ACD = anterior chamber depth; AL = axial length; BCVA = best-corrected visual acuity; CCT = central corneal thickness; ChT = choroidal thickness; D = diopters; IOP = intraocular pressure; logMAR = logarithm of the minimum angle of resolution; N = number of participants; SE = spherical equivalent; SD = standard deviation; ― = not specified due to risk of unmasking.

**Table 2:** Changes in refractive and visual parameters over six months based on linear mixed models.

	Treatment Groups		
Measurement	Placebo †	Low-Dose (0.01% Atropine) ‡	Loading Dose (0.1% to 0.01% Atropine) ‡
**AL, mm**			
Baseline §	24.60	―	―
3-month change	0.10 (0.07 to 0.13)	−0.03 (−0.06 to 0.00)	−0.08 (−0.12 to −0.05)
*p* value/adj-*P* value ||	NA	0.05/0.12	<0.001/<0.001
6-month change	0.21 (0.16 to 0.25)	−0.06 (−0.11 to −0.01)	−0.13 (−0.18 to −0.07)
*p* value/adj-*P* value ||	―	0.02/0.06	<0.001/<0.001
**SE, D**			
Baseline §	−2.99	―	―
3-month change	−0.20 (−0.32 to −0.07)	0.17 (0.03 to 0.31)	0.34 (0.20 to 0.48)
*p* value/adj-*P* value ||	―	0.02/0.05	<0.001/<0.001
6-month change	−0.37 (−0.52 to −0.21)	0.16 (−0.02 to 0.34)	0.40 (0.22 to 0.57)
*p* value/adj-*P* value ||	―	0.08/0.16	<0.001/<0.001
**Photopic pupil size, mm**			
Baseline §	2.73	―	―
3-month change	0.02 (−0.23 to 0.27)	0.18 (−0.11 to 0.48)	1.85 (1.55 to 2.15)
*p* value/adj-*P* value ||	―	0.22/0.35	<0.001/<0.001
6-month change	−0.06 (−0.28 to 0.16)	0.20 (−0.06 to 0.45)	1.83 (1.58 to 2.09)
*p* value/adj-*P* value ||	―	0.13/0.23	<0.001/<0.001
**Mesopic pupil size, mm**			
Baseline §	4.13	―	―
3-month change	0.09 (−0.22 to 0.39)	0.29 (−0.06 to 0.64)	2.02 (1.67 to 2.38)
*p* value/adj-*P* value ||	―	0.10/0.20	<0.001/<0.001
6-month change	−0.06 (−0.35 to 0.24)	0.42 (0.08 to 0.76)	2.18 (1.84 to 2.52)
*p* value/adj-*P* value ||	―	0.02/0.04	<0.001/<0.001
**Accommodation amplitude, D**			
Baseline §	16.39	―	―
3-month change	−1.54 (−3.05 to −0.04)	−1.12 (−2.75 to 0.51)	−5.72 (−7.35 to −4.09)
*p* value/adj-*P* value ||	―	0.18/0.30	<0.001/<0.001
6-month change	−2.08 (−3.59 to −0.56)	−0.45 (−1.95 to 1.04)	−4.88 (−6.37 to −3.38)
*p* value/adj-*P* value ||	―	0.55/0.75	<0.001/<0.001
**Distance BCVA, logMAR**			
Baseline §	−0.10	―	―
3-month change	0.00 (−0.02 to 0.02)	0.00 (−0.02 to 0.02)	−0.04 (−0.06 to −0.01)
*p* value/adj-*P* value ||	―	0.92/>0.99	< 0.01/0.01
6-month change	−0.01 (−0.03 to 0.01)	0.02 (−0.01 to 0.04)	−0.01 (−0.03 to 0.02)
*p* value/adj-*P* value ||	―	0.21/0.34	0.46/0.67
**Near BCVA, logMAR**			
Baseline §	−0.07	―	―
3-month change	−0.02 (−0.05 to 0.01)	0.03 (−0.01 to 0.06)	0.04 (0.01 to 0.07)
*p* value/adj-*P* value ||	―	0.11/0.20	0.02/0.06
6-month change	−0.02 (−0.05 to 0.01)	0.02 (−0.01 to 0.06)	0.04 (0.00 to 0.07)
*p* value/adj-*P* value ||	―	0.17/0.30	0.05/0.11
**IOP, mmHg**			
Baseline §	16.1	―	―
3-month change	−0.2 (−1.5 to 1.0)	0.5 (−0.9 to 1.8)	1.5 (0.2 to 2.9)
*p* value/adj-*P* value ||	―	0.49/0.68	0.03/0.06
6-month change	0.8 (−0.4 to 1.9)	−0.7 (−2.1 to 0.6)	0.0 (−1.3 to 1.3)
*p* value/adj-*P* value ||	―	0.26/0.38	>0.99/>0.99

**Notes:** Changes in refractive and visual parameters from baseline to six-month visit. All estimates are derived from constrained linear mixed models with inherent baseline adjustments. Changes in the placebo group are presented as mean change from baseline (95% CI), while changes in the 0.01% atropine and 0.1% atropine loading dose groups are presented as differences from the placebo group as mean (95% CI). **Footnotes**: †, presented as mean change from baseline (95% CI); ‡, presented as differences from the placebo group as mean (95% CI); §, the baseline value was the same for all groups; ||, adjusted for false discovery rate. **Abbreviations**: ACD = anterior chamber depth; adj-*P* = adjusted *P*; AL = axial length; BCVA = best-corrected visual acuity; CI = confidence interval; D = diopters; IOP = intraocular pressure; logMAR = logarithm of the minimum angle of resolution; SE = spherical equivalent.

**Table 3:** Adverse reactions and events.

	Treatment Groups		
	Placebo	Low-Dose (0.01% Atropine)	Loading Dose (0.1% to 0.01% Atropine)
**At 3-month visit**			
N	32	32	33
Total	6 (18.8)	8 (25.0)	22 (66.7) ***
Mydriasis	0	0	11 (33.3) **
Photophobia	0	3 (9.4)	16 (48.5) ***
Blurred near vision	0	0	18 (54.5) ***
Discomfort during administration	2 (6.3)	2 (6.3)	3 (9.1)
Other adverse reactions or events	4 (12.5)	5 (15.6)	3 (9.1)
Serious adverse reactions	0	0	0
**At 6-month visit**			
N	31	32	33
Total	4 (12.9)	1 (3.1)	15 (45.5) *
Mydriasis	0	0	7 (21.2) *
Photophobia	0	0	11 (33.3) **
Blurred near vision	0	1 (3.1)	12 (36.4) ***
Discomfort during administration	2 (6.5)	1 (3.1)	2 (6.1)
Other adverse reactions or events	3 (9.7)	0	2 (6.1)
Serious adverse reactions	0	0	0

**Notes:** Distribution of adverse reactions and events. Data are presented as the number of participants with percentages in parenthesis. Total refers to the number of participants with one or more adverse events. Investigators performed the distinction between adverse reactions and events. A further detailed reporting of adverse reactions and events was not possible due to the intention of maintaining masking. *p* values were adjusted with the false discovery rate (FDR) method and referred to as adj-*P*. **Footnotes:** *, adj-*P* < 0.05; **, adj-*P* < 0.01; ***, adj-*P* < 0.001. **Abbreviations:** FDR = false discovery rate; N = number of participants.

## Data Availability

The anonymized datasets from the current study are available from the corresponding author on reasonable request.

## Supplementary Materials

The following supporting information can be downloaded at: https://www.mdpi.com/article/10.3390/jpm13020325/s1, Table S1: Scheduled visits and examinations; Table S2: Parental self-reported weekly compliance.
