# Supplementary material for: Myopia Control with Low-Dose Atropine in European Children: Six-Month Results from a Randomized, Double-Masked, Placebo-Controlled, Multicenter Study
Source: J Pers Med. 2023 Feb 14;13(2):325. doi: 10.3390/jpm13020325 (PMC9960354; doi:10.3390/jpm13020325)
Supplement: Supplementary file 1 [file jpm-13-00325-s001.zip › jpm-2189172-supplementary.pdf]

Myopia Control with Low-Dose Atropine in European Children: Six-Month Results from a Randomized, Double-Masked, Placebo-Controlled, Multicenter Study

Anders Hvid-Hansen [1,*], Nina Jacobsen [1,2], Flemming Møller [3], Toke Bek [4], Brice Ozenne [5,6] and Line Kessel [1,2]

[1] Department of Ophthalmology, Copenhagen University Hospital—Rigshospitalet-Glostrup, DK-2600 Glostrup, Denmark
[2] Department of Clinical Medicine, University of Copenhagen, DK-2200 København N, Denmark
[3] Department of Ophthalmology, University Hospital of Southern Denmark—Vejle Hospital, DK-7100 Vejle, Denmark
[4] Department of Ophthalmology, Aarhus University Hospital, DK-8200 Aarhus N, Denmark
[5] Department of Public Health, Section of Biostatistics, University of Copenhagen, DK-1014 København K, Denmark
[6] Neurobiology Research Unit, Copenhagen University Hospital—Rigshospitalet, DK-2200 København N, Denmark
* Correspondence: anders.johan.hvid-hansen@regionh.dk

Table S1: Scheduled visits and examinations

	Scree	Base	3 mo	6 mo	9 mo	12 mo	18 mo	24 mo	30 mo	36 mo
Slit lamp exam	X									
Dilated fundus exam	X									
Non-cycloplegic autoref.	X	X	X	X	X	X	X	X	X	X
Cycloplegic autoref.	X	X	X	X	X	X	X	X	X	X
Manifest refraction	X	X	X	X	X	X	X	X	X	X
Distance BCVA	X	X	X	X	X	X	X	X	X	X
Near BCVA		X	X	X	X	X	X	X	X	X
Accommodation amplitude		X	X	X	X	X	X	X	X	X
IOLMaster 700	X	(X)	X	X	X	X	X	X	X	X
Scheimpflug imaging	X	(X)	X	X	X	X	X	X	X	X
Pupillometry	X	(X)	X	X	X	X	X	X	X	X
SS-OCT		X	X	X	X	X	X	X	X	X
Intraocular pressure	X	(X)	X	X	X	X	X	X	X	X
Trial medication		X	X	X	X	X	X	X		
Compliance										
Registration of AR and AE		X	X	X	X	X	X	X	X	X

Footnotes: (X), optional examination if the period between screening and baseline was less than four weeks.

Abbreviations: AE = adverse events; AR = adverse reaction; autoref. = autorefraction; Base = Baseline; BCVA = best-corrected visual acuity; mo = months; Scree = screening; SS-OCT = swept-source optical coherence tomography.

Table S2: Parental self-reported weekly compliance

Compliance, N (%)	Treatment groups		
	Placebo	Low-dose (0.01% atropine)	Loading dose (0.1% to 0.01% atropine)
**At 3-month visit**			
N	32	32	33
6 days	1 (3.1)	1 (3.1)	3 (9.1)
7 days	31 (96.9)	31 (96.9)	30 (90.9)
**At 6-month visit**			
N	31	32	33
6 days	1 (3.2)	1 (3.1)	3 (9.1)
7 days	30 (96.8)	31 (96.9)	30 (90.9)

Notes: Weekly compliance as reported by the parents and presented as the number of days the trial medication was administered as prescribed during an average week since the last follow-up. No parents reported compliance below six days a week.

Footnotes: *, adj-P < 0.05; **, adj-P < 0.01; ***, adj-P < 0.001.

Abbreviations: N = number of participants.
